# EAES Recommendations for Recovery Plan in Minimally Invasive Surgery Amid COVID-19 Pandemic

**DOI:** 10.1007/s00464-020-08131-0

**Published:** 2020-11-10

**Authors:** Alberto Arezzo, Nader Francis, Yoav Mintz, Michel Adamina, Stavros A. Antoniou, Nicole Bouvy, Catalin Copaescu, Nicolò de Manzini, Nicola Di Lorenzo, Salvador Morales-Conde, Beat P. Müller-Stich, Felix Nickel, Dorin Popa, Diana Tait, Cenydd Thomas, Susan Nimmo, Dimitrios Paraskevis, Andrea Pietrabissa, The EAES Group of Experts for Recovery Amid COVID-19 Pandemic, Muriel Eck, Emina Letić, Silviu Daniel Preda, Alice Tsai, Ewelina Malanowska, Dusan Lesko, Wlodzimierz Majewski, Ludovica Baldari, Luca Morelli, Andreas Shamiyeh, Gil Faria, Francesco Maria Carrano, Piotr Mysliwiec, Gunnar Ahlberg, Elisa Cassinotti, Samir Delibegović, Lubomír Martinek, Eugenia Yiannakopoulou, Marguerite Gorter-Stam, Marguerite Gorter-Stam, George Hanna, Hans Fuchs, Miloš Bjelovic, Sheraz Markar, Philip Wai Yan, Chiu, Bang Wool Ecom, Young-Woo Kim, Carmen Balagué Ponz, Marlies Schijven, Luigi Boni, Thomas Carus, George Theodoropoulos, Antonello Forgione, Marco Milone, Wanda Luisa Rita Petz, Predag Andrejevic, Dejan Ignjatovic, Thanjakumar Arulampalam, Kenneth Campbell, Manish Chand, Mark Coleman, Christos Kontovounisios, Chen Sagiv, F anny Ficuciello, Stefania Marconi, P ietro Mascagni, K iyokazu Nakajima, Francisco Miguel Sánchez Margallo, Tim Horeman, George Mylonas, Pietro Valdastri

**Affiliations:** 1 Department of Surgical Sciences, University of Torino, Corso AM Dogliotti 14, 10126 Torino, Italy; 2 Department of General Surgery, Yeovil District Hospital NHS Foundation Trust, Yeovil, UK; 3 The Griffin Institute, The Northwick Park Institute for Medical Research, Northwick Park and St Marks Hospital, Watford Road, Harrow, Middlesex, London, HA1 3UJ UK; 4 Department of Surgery, Hadassah, Hebrew University Medical Center, Jerusalem, Israel; 5 Department of Surgery, Clinic of Visceral and Thoracic Surgery, Cantonal Hospital Winterthur, Winterthur, 8401 Zurich, Switzerland; 6 Faculty of Medicine, University of Basel, 4051 Basel, Switzerland; 7 Medical School, European University Cyprus, Nicosia, Cyprus; 8 Department of Surgery, Mediterranean Hospital of Cyprus, Limassol, Cyprus; 9 Department of Surgery, Maastricht University Medical Centre, Maastricht, The Netherlands; 10 Department of Gastrointestinal and Bariatric Surgery, Ponderas Academic Hospital, Bucharest, Romania; 11 General Surgery Clinic, Department of Medical, Surgical and Health Sciences, University of Trieste, University Hospital of Trieste, Trieste, Italy; 12 Department of Surgical Sciences, University of Rome “Tor Vergata”, Rome, Italy; 13 Unit of Innovation in Minimally Invasive Surgery, Department of General and Digestive Surgery, University Hospital “Virgen del Rocio”, University of Sevilla, Sevilla, Spain; 14 Department of General, Visceral and Transplantation Surgery, Heidelberg University Hospital, Heidelberg, Germany; 15 General Surgery, Linköping University Hospital, Linköping, Sweden; 16 The Royal Marsden NHS Foundation Trust, London, UK; 17 Department of Radiology, Yeovil District Hospital NHS Foundation Trust, Higher Kingston, Yeovil, UK; 18 Department of Anaesthesia, Critical Care and Pain Medicine, Western General Hospital, Edinburgh, Scotland; 19 Department of Hygiene, Epidemiology and Medical Statistics, Medical School, National and Kapodistrian University of Athens, Athens, Greece; 20 Department of Surgery, Fondazione IRCCS Policlinico San Matteo, Pavia, Italy

**Keywords:** COVID-19, Delphi consensus, EAES guidance, Priority, Minimally invasive surgery

## Abstract

**Background:**

COVID-19 pandemic presented an unexpected challenge for the surgical community in general and Minimally Invasive Surgery (MIS) specialists in particular. This document aims to summarize recent evidence and experts’ opinion and formulate recommendations to guide the surgical community on how to best organize the recovery plan for surgical activity across different sub-specialities after the COVID-19 pandemic.

**Methods:**

Recommendations were developed through a Delphi process for establishment of expert consensus. Domain topics were formulated and subsequently subdivided into questions pertinent to different surgical specialities following the COVID-19 crisis. Sixty-five experts from 24 countries, representing the entire EAES board, were invited. Fifty clinicians and six engineers accepted the invitation and drafted statements based on specific key questions. Anonymous voting on the statements was performed until consensus was achieved, defined by at least 70% agreement.

**Results:**

A total of 92 consensus statements were formulated with regard to safe resumption of surgery across eight domains, addressing general surgery, upper GI, lower GI, bariatrics, endocrine, HPB, abdominal wall and technology/research. The statements addressed elective and emergency services across all subspecialties with specific attention to the role of MIS during the recovery plan. Eighty-four of the statements were approved during the first round of Delphi voting (91.3%) and another 8 during the following round after substantial modification, resulting in a 100% consensus.

**Conclusion:**

The recommendations formulated by the EAES board establish a framework for resumption of surgery following COVID-19 pandemic with particular focus on the role of MIS across surgical specialities. The statements have the potential for wide application in the clinical setting, education activities and research work across different healthcare systems.

**Electronic supplementary material:**

The online version of this article (10.1007/s00464-020-08131-0) contains supplementary material, which is available to authorized users.

The rapid spread of the coronavirus disease 2019 (COVID-19) presents an unprecedented crisis to the surgical community globally [1, 2]. During the pandemic, elective surgical practice was forced to rapidly decrease or even put on hold completely. As a consequence, there is a backlog of patients requiring surgical services but with limited human and institutional resources [3–8].

While health care systems are calling to resume elective surgical practice where pandemic is under control [1, 2], uncertainty remains about the duration of the pandemic, the possibility of a second wave and the extent of its consequences on surgical services and patients [9, 10]. This leaves the surgical community with unanswered clinical questions on patient and staff safety.

Various guidelines and recommendations have been published on issues pertaining to adapting surgical practice during the pandemic, often with conflicting recommendations, whereas many may not have been based on research evidence [11–14]. Preliminary guidelines recommended against performing laparoscopic surgery to avoid putative risks of SARS-CoV2 transmission via aerosolization due to pneumoperitoneum but were later revised recommending laparoscopic surgery under restrictions [15]. Several subsequent guidelines did not recommend against laparoscopic surgery; however, they advised strict precautions such as closed circuit smoke evacuation and the use of filtering system and personal protective equipment (PPE) [16–19]. The role of MIS has been argued to be more favourable in SARS-CoV-2 positive patients, as the potential benefits of MIS might exceed the risk of pneumoperitoneum on cardiovascular and respiratory systems [20].

Furthermore, there is substantial uncertainty with regard to specific questions related to laparoscopic procedures, and structured guidance pertinent to MIS procedures is lacking. There is an urgent need for practice-based recommendations in specific clinical situations with regard to safety precautions for patients and staff amidst the COVID-19 pandemic [21].

The purpose of this EAES initiative was to generate an updated and comprehensive set of management recommendations for MIS for each subspeciality of general surgery. This consensus was also designed to document the broader experts’ opinion on how to optimize the use of human and institutional resources including the use of MIS techniques for patient benefit.

## Methods

A steering group (SG) was formed comprising of six experts from the EAES Executive Board (AA, NF, YM, SM, DP and AP) who organized the project and guided the data synthesis. IRB approval and informed written consent were not required. The steps of the consensus process are outlined below.

### Identification of domains and formulation of questions

The clinical questions were divided into eight domains: (i) general (ii) Hepatobiliary & Pancreatic (HPB), (iii) Bariatrics (B), (iv) Abdominal Wall (AW), (v) Endocrine (E), (vi) Upper Gastrointestinal (UGI), (vii) Lower Gastrointestinal (LGI), (viii) technology and research. Domains (ii) to (vii) were divided into two subdomains: emergency and elective.

### Identification of experts to address questions

Fifty-Five out of EAES board members accepted the task to contribute to this project and were divided into eight subgroups organized by the domain topics and led by a designated chair (Table 1). In addition, key stakeholders were invited to provide expert input into the multidisciplinary aspects of this project including anaesthesiology, radiology and oncology and were allocated to a relevant group, based on their expertise.

**Table 1:** Expert group members and topic allocations

Topic	Leader	Experts
General introduction	Catalin Copaescu	Emina Letić, Silviu Daniel Preda, Alice Tsai, Ewelina Malanowska, Dusan Lesko, Wlodzimierz Majewski, Ludovica Baldari
Hepatobiliary	Nicolò de Manzini	Luca Morelli, Andreas Shamiyeh, Gil Faria
Bariatrics	Nicola Di Lorenzo	Francesco Maria Carrano, Piotr Mysliwiec, Gunnar Ahlberg
Abdominal Wall	Stavros Antoniou	Elisa Cassinotti, Samir Delibegović, Lubomír Martinek
Endocrine	Nicole Bouvy	Eugenia Yiannakopoulou, Marguerite Gorter-Stam, Hendrik Jaap Bonjer
Upper GI	Beat P. Müller-Stich	George Hanna, Hans Fuchs, Miloš Bjelovic, Sheraz Markar, Philip Wai Yan, Chiu, Bang Wool Eom, Young-Woo Kim, Carmen Balagué Ponz, Marlies Schijven
Lower GI	Michel Adamina	Luigi Boni, Thomas Carus, George Theodoropoulos, Antonello Forgione, Marco Milone, Wanda Luisa Rita Petz, Predag Andrejevic, Dejan Ignjatovic, Thanjakumar Arulampalam, Kenneth Campbell, Manish Chand, Mark Coleman, Christos Kontovounisios
Technology & Research	Felix Nickel	Chen Sagiv, Fanny Ficuciello, Stefania Marconi, Pietro Mascagni, Kiyokazu Nakajima, Francisco Miguel Sánchez Margallo, Tim Horeman, George Mylonas, Pietro Valdastri

### Search methods and inclusion criteria

Based on the research questions, a literature search was designed and performed by two independent EAES experts (AA, NF). The PubMed and Embase databases were queried for articles published before May 10th, 2020. Inclusion criteria were systematic and narrative reviews, commentaries, randomized clinical trials, cohort studies and case series on the subject of surgery during the COVID-19 and other pandemic published in the English language. Search syntaxes used was (COVID OR ‘SARS CoV 2′ OR coronavirus AND surgery).

### Formulation of questions and statements

Questions were drafted and submitted to the steering group, which approved them prior to sending them to the subgroups to formulate statements in response to these questions. Statements were generated by each subgroup in each topic question of their domain. Each group was advised to include any available evidence to support their statements and when evidence was not available, experts’ opinion was considered. Each group conducted a literature research and drafted statements and recommendations in response to their research questions. The literature review was reported in accordance with PRISMA statement standards for systematic reviews and meta-analyses [22]. Statements were then submitted to the steering group (SG) who did not participate in the formulation of these statements or in the voting process. After structural editing by the SG group, all questions were unanimously approved by all experts (Table 1).

### Voting and data analysis

The statements received from each subgroup of experts was compiled by the SG and a modified Delphi methodology process was followed to reach agreement among all the experts on all statements and recommendations [23].

EAES board members voted to declare agreement or disagreement with the statements using closed-ended questions (agree or disagree), whereas there was an option to submit free text comments. The Delphi process was implemented through the SurveyMonkey electronic platform (https://nl.surveymonkey.com). To reduce the possibility of bias among participants, the authors of the statements and the resultant votes/comments remained anonymous. Each statement was subjected to live voting by all experts including key stakeholders and excluding the steering committee.

Consensus was achieved when a statement reached at least 70% expert agreement. Statements with less than 70% agreement in the first round were returned to the expert subgroup who modified them according to the comments. The subgroups had the option to revise statements based on feedback for further voting.

The results of the consensus are summarized using descriptive statistics. The manuscript was then drafted with the recommendations following completion of all voting and statement formulation and sent to all members for revision, input and approval prior to submission for publication.

## Results

Ninety-two statements were generated by the subgroups across the eight topics. The full text literature analyses and references used to generate statements and recommendations for all topics are included as Appendix 1.

Eighty-four out of 92 statements (91%) were approved in the first round and further 8 modified statements were approved in the second round.

Overall, the Delphi process approved 92 statements (100%) for the consensus guidelines. The questions, final recommendations and respective approval rates in each step of the Delphi processes are summarized in Table 2.

**Table 2:** Questions and statements

Topics	Questions	Statement	1st round % YES	2nd round % YES
Introductory questions	1. What are the appropriate measures to mitigate the risks of general anaesthesia in patients during COVID- 19 pandemic?	1. All patients requiring surgery under general anaesthesia should be tested for COVID-19 by means of RT-PCR	79%	
Patient safety	a. Asymptomatic	2. In case of PCR testing not available, imaging modalities such as CT or US lung scan can be used as a diagnostic tool before general anaesthesia	90%	
	b. Symptomatic	3. Surgical procedures should be carefully prioritized based on local resources, the regional control of the COVID-19 pandemic and the patients’ medical condition	65%	98%
		4. When indicated, emergency surgery should be performed in all patients regardless of their COVID-19 status	94%	
		5. In COVID-19 positive patients, elective surgery for cancer and progressive diseases should be considered only after a negative PCR COVID-19 test	80%	
		6. In case of overutilization of hospital resources alternative/holding oncological therapies could be proposed to treat cancer in COVID-19 negative patients	80%	
		7. In case of symptomatic patients either suspected or confirmed COVID-19 positive, regional anaesthesia techniques should be considered when possible	92%	
Introductory questions	2. What is the optimal surgical approach during COVID- 19 pandemic in COVID + o symptomatic?		94%	
Surgical approach	a. In elective vs. emergency cases			
	b. in patients with moderately to severely compromised respiratory function (CPAP or endotracheal intubation)			
	c. in patients with mild to moderately compromised respiratory function (requiring mask oxygen therapy only)			
	d. in patients with normal respiratory function—Patients with either no symptoms or mild symptoms, without interstitial pneumonia or other pulmonary complications			
	3. What is the optimal surgical approach during COVID- 19 pandemic in COVID – or unknow but not suspected?	8. General preference for minimal invasive surgery (MIS) according to guidelines should not change both in COVID-19 negative and positive patients as well as in elective and emergency settings, unless otherwise contraindicated, if adequate equipment and expertise are available		
	a. In elective vs. emergency cases	9. No a priori contraindication to a minimally invasive approach should be stated in COVID-19 positive patients with normal, mild or moderately compromised respiratory function	92%	
Staff protection in OR	4. What is the optimal personal protection equipment (PPE) that should be used during abdominal surgery in?	10. The OR personnel should use standard PPE when operating on a tested COVID-19 negative patient	80%	
	a. asymptomatic patients during COVID- 19 pandemic			
	b. symptomatic patients during COVID- 19 pandemic			
	c. positive patients during COVID- 19 pandemic	11. When any aerosol generating procedure is indicated on a suspected or confirmed COVID-19 positive patient, symptomatic or asymptomatic, the OR staff should be reduced to minimum and should all wear high level of PPE, consisting of: medical hood, FFP2/FFP3 mask, eye protection/full-face shield, long sleeved fluid repellent gown / medical protecting coverall (ANSI/AAMI level 3–4), double disposable gloves, long waterproof leg cover	94%	
	d. in symptomatic or positive patients and Aerosol Generating Procedures (Laparoscopy/Endoscopy) during COVID- 19 pandemic	12. It is of major importance that donning and doffing is performed under self-control or direct control of a colleague	85%	
Specific measures in OR	5. Which Specific Operative Risk Issues to consider during abdominal surgery in symptomatic or positive patients during COVID- 19 pandemic regarding?	13. The risk of infection during laparoscopic surgery should be controlled by reducing gas leaks, the generation of smoke and by the application of surgical smoke evacuating systems	98%	
	a. Reduce the risk of aerosol contamination during laparoscopy	14. In order to control gas leaks, surgeons should: use low CO_2_ intraabdominal pressure, perform small incisions for the access ports, limit the exchange of surgical instruments and evacuate CO_2_ before any abdominal wall incision	94%	
	b. Minimizing Staff personnel	15. In order to reduce the smoke generation during surgery in COVID-19 positive or suspected patients, the application of energy devices should be minimized, whereas ligatures/clips and/or stapling devices should be considered instead	65%	87%
	c. Type of OR – COVID + OR/cleaning OR after surgery/negative pressure OR	16. COVID-19 positive or suspected patients should be operated in a dedicated OR equipped with laminar air flow, negative pressure and downward evacuation system which should be cleaned by a dedicated specifically trained 24/7 team	90%	
	6. How should we change the design of the OR block to adapt to the risks of infections in the era of COVID- 19 pandemic?	17. Disposable devices (instruments, trocars, etc.) should be preferred in COVID-19 positive or suspected patients	76%	
	a. Negative pressure	18. All patients admitted should follow an initial screening triage, considering history of the patient, temperature, nasal swab and chest radiogram to detect COVID-19 status	73%	
	b. Distinct paths for COVID + and – patients	19. Based on screening at admission, patients should be addressed to the COVID-19 positive, COVID-19 negative or suspected unit, as for local organization	94%	
	c. Design of equipment easily disinfected or disposable covers	20. Distinct and separated paths should be created in any hospital for COVID-19 positive, COVID-19 negative or suspected patients	98%	
Specific emergency operations in the immediate post-COVID-19 pandemic				
Hepatobiliary	7. What are specific measures in hepatobiliary disorders that should be considered during COVID-19 pandemic regarding?	21. Antibiotics should be attempted as the only treatment for COVID positive/ suspected patients with severe cholecystitis and the response to treatment should be reassessed rapidly (24 h)	83%	
	a. Role of a non-operative approach (antibiotics) in cholecystitis	22. Transhepatic drainage should be proposed for compromised patients with severe cholecystitis, refractory to medical treatment at 24 h in COVID-19 positive patients	78%	
	b. Role of a non-operative approach (transhepatic drainage) in cholecystitis	23. In COVID-19 positive patients with common bile duct and gall bladder stones a sequential approach (ERCP followed by Laparoscopy) should be preferred to a Laparo-Endoscopic Rendez-Vous (LERV) approach to reduce the risk of a prolonged anaesthesia	84%	
	c. Role of a Laparo-Endoscopic Rendez-Vous (LERV) approach (orotracheal intubation) in Jaundice for CBD obstruction	24. Patients with obstructive common bile duct stones should be treated according to the severity of cholangitis regardless of the COVID-19 status, favouring medical treatment	77%	
	d. Role of non-surgical/ non-endoscopic approach (only ERCP) in Jaundiced patients	25. Patients with a non-calcular obstructive jaundice should be referred to tertiary centres in order to choose the best treatment (PTBD-ERCP-upfront surgery)	84%	
		26. Upfront surgery should not be offered to COVID-19 positive patients with non-calcular obstructive jaundice	80%	
		27. Cholecystectomy should be indicated in severe cholecystitis that is not responsive to conservative or interventional treatment, even COVID-19 positive patients	89%	
Abdominal wall hernias	8. What are specific measures in abdominal wall hernia surgery that should be considered during COVID-19 pandemic regarding?	28. Laparoscopic approach to incarcerated ventral and inguinal hernia may be safe in COVID-19 positive patients if laparoscopy is not otherwise contraindicated	80%	
	a. Laparoscopy in incarcerated ventral/incisional hernia			
	b. Laparoscopy in incarcerated inguinal hernia			
	c. Role of meshes in emergency	29. In COVID-19 unknown patients, delaying surgery of an incarcerated ventral and inguinal hernia to obtain test results may not be justified	89%	
	d. Role of techniques that might increase intraabdominal pressure	30. The use of mesh for hernia repair may not increase the risk of complications in COVID-19 positive patients	89%	
	e. Surgical approach if a bowel resection is needed	31. Laparoscopic approach to incarcerated hernia requiring bowel resection may be safe for COVID-19 positive patients if not otherwise contraindicated	80%	
Upper GI	9. What is the role for flexible endoscopy mitigating the risk of surgery during COVID-19 pandemic in the following situations?	32. Flexible endoscopic therapy should be the first attempt to treat upper GI bleeding even in patients affected by COVID-19	93%	
	a. Acute gastric volvulus			
	b. Obstructing gastric cancer			
	c. Obstructing esophageal cancer			
	d. Esophageal perforation			
	e. Surgical leaks			
	f. Bleeding	33. COVID-19 positive patients with an obstructing esophageal or gastric cancer should be treated first by endoscopic stenting if possible, in order to delay surgery until conditions are more favourable to operate	81%	
	10. What is the role for surgical endoscopy during COVID-19 pandemic in the following situations?	34. COVID-19 positive patients with an immediate presentation of benign esophageal perforation (of less than 24 h) should be treated first by flexible endoscopy means, while those perforated present after 24 h should undergo immediate surgery	83%	
	a. Esophageal perforation	35. In patients suffering from an upper GI anastomotic leak, initial endoscopic therapy should be attempted regardless the COVID-19 status	88%	
	b. Gastroduodenal perforation	36. Emergency surgery should be recommended after failure of conservative/endoscopic management in symptomatic upper GI perforation or leak in COVID-19 positive patients	98%	
	c. Bleeding	37. Laparoscopy should be the preferred approach in patients with perforated gastroduodenal ulcer if not otherwise contraindicated in COVID-19 positive patients	84%	
Lower GI	11. What is the role for flexible endoscopy mitigating the risk of surgery during COVID-19 pandemic in the following situations?	38. Endoscopic stenting for obstructing colorectal carcinoma should be considered for palliation in malignant obstruction regardless of the COVID-19 status	67%	87%
	a. Stenting for obstructing colorectal carcinoma			
	b. Decompression of acute sigmoid volvulus			
	c. Management of acute perforation	39. Endoscopic decompression should be the first line of treatment of uncomplicated sigmoid volvulus, regardless the COVID-19 status	91%	
	d. Management of anastomotic leaks	40. In patients suffering from a leak of a low rectal anastomosis, all endoscopic means which proved effective should be attempted regardless the COVID-19 status	81%	
	12. What is the role for laparoscopy during COVID-19 pandemic in the following situations?	41. Emergency surgery should be recommended after failure of conservative/endoscopic management in symptomatic lower GI perforation or leak in COVID-19 positive patients	93%	
	a. Acute diverticulitis (lavage/HP/Primary Resection anastomosis with stoma)	42. The indication for laparoscopic lavage for Diverticular Disease can be considered in COVID-19 positive patients when local expertise and protective measures are available	65%	87%
	b. Small bowel obstruction	43. Primary resection with or without anastomosis can be considered in COVID-19 positive patients with acute diverticulitis, providing that this is performed by an experienced surgeon	86%	
	c. Appendicitis (with/without abscess/abdominal collection)	44. Percutaneous drainage and/or targeted defunctioning stoma can be considered in unstable COVID-19 positive patients with acute diverticulitis	88%	
	13. Should damage control surgery be performed in COVID + / suspected COVID-19 patients?	45. Laparoscopic approach should be considered in COVID-19 positive patients with virgin abdomen and acute small bowel obstruction that is likely due to a single adhesion band, which is suspected at CT scan	91%	
	a. In Upper GI	46. Non-surgical approaches such as percutaneous drainage with antibiotic treatment should be considered as the first line of treatment of acute appendicitis with peri-appendicular abscess in COVID-19 positive patients	81%%	
	b. In Lower GI	47. In case of failed non-surgical approach for acute appendicitis in COVID-19 positive patients, laparoscopic surgery should be considered	91%	
	c. In Pancreas surgery	48. The principles of damage control surgery with adherence to optimal seal of temporary abdominal closure should remain unchanged in COVID-19 positive/suspected patients	98%	
Specific elective operations in the immediate post-COVID-19 pandemic				
Hepatobiliary	14. What indications for laparoscopic surgery in hepatobiliary disorders should be considered during COVID-19 pandemic regarding?	49. Elective cholecystectomy in patients COVID-19 negative may be performed if hospital setting allows a safe pathway for COVID-19 negative patients and local resources are sufficient	98%	
	a. Cholelithiasis/choledocholithiasis and adenomyoma	50. Elective cholecystectomy in patients COVID-19 positive should be delayed until the post-pandemic period	81%	
	b. Hepatic cancer	51. Elective liver resection for primary or secondary cancer in COVID-19 negative patients should not be delayed	86%	
	c. Liver metastases	52. Elective liver resection for primary or secondary cancer in COVID-19 positive patients should be delayed until patients fully recover from COVID. Jaundice or infection should be first treated with PTBD or ERCP as a bridge therapy	93%	
	d. Pancreatic cancer	53. Patients affected by unproven pancreatic lesion could be observed and intervention can be delayed until the post-pandemic period	69%	73%
	e. Other indications	54. Elective pancreatic resection for cancer in COVID-19 negative patients should not be delayed	90%	
		55. Elective liver resection for cancer in COVID-19 positive patients should be delayed until patients fully recover from COVID. Jaundice or infection should be first treated with PTBD or ERCP as a bridge therapy	90%	
Bariatrics	15. What is the role for flexible endoscopy mitigating the risk of surgery during COVID-19 pandemic in bariatrics?	56. In order to mitigate the risk of surgery during the recovery plan after the COVID-19 pandemic, all endoscopic techniques for morbid obesity should be preferred as bridge to surgery	70%	
	a. Should bariatric procedure be postponed?			
	b. Role of bridging procedures (balloons and others)			
	c. Treatment of complications of bariatric surgery	57. Intragastric Balloon placement can be a valid alternative to endoscopic sleeve gastroplasty in order to reduce procedure times and resources usage during the COVID pandemic	68%	76%
	16. Is there a different timing for surgery during COVID-19 pandemic in bariatrics?	58. In patients experiencing a complication (bleeding or leak) following a bariatric procedure, all endoscopic means which proved effective should be put in place regardless of COVID-19 status	98%	
	a. Should bariatric procedure be postponed?	59. During the recovery plan after the COVID-19 pandemic, bariatric surgery should not be postponed further, nor the indications limited in areas with a low incidence of SARS-CoV-2 infections	77%	
	b. When should a redo procedure be performed?	60. In case local regulatory authorities impose a reduction of bariatric weekly case load, more complex metabolic patients should be prioritized	98%	
Abdominal wall	17. What indications to surgery and what anaesthesia in abdominal wall disorders should be considered during COVID-19 pandemic regarding?	61. Elective laparoscopic treatment for ventral and inguinal hernias in COVID-19 negative asymptomatic or poorly symptomatic patients may need to be postponed	90%	
	a. Ventral hernias			
	b. Inguinal hernias			
	c. Role of watch&wait policy	62. A watchful waiting management may be safe in asymptomatic patients and patients with abdominal wall hernia and minimal symptoms that do not substantially affect quality of life	90%	
	d. Should Spinal anaesthesia always be preferred? what will be the indications?	63. Both general and spinal anaesthesia for hernia repair should be considered safe in COVID-19 negative patients. The choice should follow local guidelines and patient’s preference	95%	
Endocrine	18. What indications to laparoscopic surgery in endocrine disorders should be considered during COVID-19 pandemic regarding?	64. In the recovery plan after COVID-19 Pandemic indications to laparoscopic surgery and priorities in endocrine disorders should not change	95%	
	a. Functional adrenal tumour	65. Elective adrenal resection for primary or secondary cancer in COVID-19 negative patients should not be delayed	98%	
	b. Adrenal cancer	66. Elective adrenal resection for primary or secondary cancer in COVID-19 positive patients should be delayed until patients fully recover from COVID	93%	
	c. Adrenal metastases	67. Elective adrenal resection for functional tumours (pheochromocytoma and severe Cushing) in COVID-19 negative patients should not be delayed	93%	
	d. Thyroid goiter	68. Elective adrenal resection for functional tumours (pheochromocytoma and severe Cushing) in COVID-19 positive patients should be delayed until patients fully recover from COVID	93%	
	e. Thyroid cancer (or suspected)	69. Elective surgery for thyroid nodules Bethesda V and TIRADS 5 or higher in COVID-19 negative patients should not be delayed	95%	
	f. P-Nets	70. Elective surgery for thyroid nodules Bethesda V and TIRADS 5 or higher in COVID-19 positive patients should be delayed until patients fully recover from COVID	95%	
		71. Patients affected by thyroid goiter severely symptomatic for dyspnea should not have surgery delayed regardless of the COVID status	93%	
Upper GI	19. When and how should surgery for Upper GI disorders be postponed during COVID-19 pandemic?	72. In COVID-19 positive patients who suffer from early esophageal or gastric cancer surgery should be delayed and neoadjuvant treatment should be considered even if not normally indicated	71%	
	a. Should we extend the indications for neoadjuvant treatment to early esophageal cancer?			
	b. Should we extend the duration (cycles) of neoadjuvant treatment to esophageal cancer?			
	20. What is the role for flexible endoscopy mitigating the risk of surgery during COVID-19 pandemic in upper GI functional diseases?			
	a. Achalasia (including POEM)?			
	b. Anti-reflux procedures (Esophyx/MUSE)?			
	21. Does surgical endoscopy still have a role during COVID-19 pandemic in the treatment of the following diseases?			
	a. Esophageal cancer	73. Patients affected by benign Upper GI functional disorders such as achalasia and GERD should be considered for flexible endoscopic intervention if not responding to medical treatment, after the COVID pandemic	59%	93%
	b. Gastric cancer (total / subtotal gastrectomy)	74. Patients affected by neoplastic disease of the upper GI for whom surgery is indicated, should be considered for an MIS approach, if not otherwise contraindicated, after the COVID pandemic	81%	
	c. Hiatal hernia & reflux disease	75. In COVID-19 positive patients with a surgical indication for GERD, hiatal hernia or achalasia surgery should be delayed	88%	
Lower GI	22. When and how should surgery for Lower GI disorders be postponed during COVID-19 pandemic?	76. Prioritization of elective surgery for benign colorectal pathologies should be made taking into account the patient and disease characteristics, local COVID-19 burden and institutional and staff resources	95%	
	a. Should we extend the indications for neoadjuvant treatment to early rectal cancer?			
	b. Should be opt for total neoadjuvant radiochemotherapy including upfront chemotherapy, for rectal cancer?			
	c. How much should we wait after CRT?			
	d. Role of watch&wait policy for rectal cancer with complete response			
	e. Should we propose total neoadjuvant chemotherapy for colon cancer?			
	f. Should we extend the indications for “liver first” and postpone rectal resection?			
	23. What is the risk in performing colorectal anastomoses during COVID-19 pandemic?	77. Neoadjuvant therapy could be considered in early rectal cancer in order to postpone surgery after the COVID pandemic, within registered studies	66%	82%
	a. Should we avoid anastomosis (HP?)	78. Patients with rectal cancer should not be offered chemoradiotherapy including upfront chemotherapy as a sole treatment outside clinical trials	83%	
	b. Should always protect anastomosis with stoma?	79. Surgery for rectal cancer in patients COVID-19 positive should be delayed beyond the standard 12 weeks following neoadjuvant chemoradiotherapy	78%	
	24. What is the role for transanal surgery during COVID-19 pandemic?	80. A watch and wait policy for neoadjuvant treated rectal cancer should be proposed only in the setting of clinical trials and/or when surgery is contraindicated	82%	
	a. Cost/effectiveness of transanal surgery compared to flexible endoscopy in terms of risk for patient and operators?	81. During the COVID-19 pandemic and the recovery plan, neoadjuvant chemotherapy can be proposed to patients affected by stage II and III colon cancers	78%	
	b. Role of TaTME and ultra-low anastomosis?	82. A liver first approach in locally advanced rectal cancer and synchronous liver metastases cannot be recommended solely based on the pandemic situation	95%	
	25. What is the optimal surgical approach during COVID- 19 pandemic for?	83. In COVID-19 negative patients undergoing elective colorectal resection, anastomosis should be considered if not otherwise contraindicated	98%	
	a. Colon cancer	84. Stoma formation should be preferred to an anastomosis in all patients medically unfit or COVID-19 positive	83%	
	b. Symptomatic chronic diverticulitis	85. Alternative strategies to TEMS/TAMIS for low rectal lesions, such as endoscopic mucosal resection and endoscopic submucosal dissection, should be considered in COVID-19 positive	72%	
	c. Inflammatory Bowel Disease	86. Overall, taTME and ultra-low anastomosis are procedures at higher risk of complications and should only be performed selectively in expert centres to minimize resource consumption during the pandemic	93%	
	d. Rectal prolapse (laparoscopy vs transanal approach)	87. MIS approach should be considered to electively treat colon cancer as well as benign conditions such as inflammatory bowel diseases and recurrent diverticulitis, due to its well proved benefits of reducing morbidity, during the pandemic	88%	
New technologies demanded				
Technology	26. Which specific Operative Risk Issues to consider in case of abdominal surgery in symptomatic or positive patients during COVID- 19 pandemic?	88. Research activity on digital technology and robotics should be encouraged to focus on reducing personnel in wards, intensive care unit and OR	98%	
	a. Use of active/passive smoke evacuator			
	b. Use of reusable/disposable trocars			
	c. Use specific type of reusable trocars (Ballon at the tip, bladeless, etc.…)			
	d. Use of reusable/disposable instruments			
	e. Use of advanced dissectors (Ultrasonic, Radiofrequency,…) vs standard mono & bipolar			
	f. Use of cold knifes, scissors and ligatures/sutures			
	27. Is it time to use robotics technology to reduce the employment of human beings and/or to keep social distance for…			
	a. OR surgical instrumentation			
	b. Scrub nurse and OR personnel			
	c. Ward personnel			
	d. Other…			
	28. In the era of attention to climate changes and pollution, how to deal with technology solutions to limit quantity of waste dispersal?			
	a. How to recycle PPE?			
	b. How to recycle the increasing disposable material?			
	c. How to be sure that reprocessing is effective?			
	29. While the COVID-19 pandemic is severely affecting educational programs in surgery, can we envision technology solutions for training, such as …?			
	a. Hands on courses on 3D printed organs / districts			
	b. Virtual Reality simulators			
	c. Consultation of selected Videolibrary			
	d. Attending of Online Congresses			
	e. Extensive webinar activity for education	89. Practical technological solutions including sustainable materials and steam sterilization for PPE should be investigated in order to minimize production of waste	96%	
	f. Real time education via telesurgery for open and laparoscopic operations	90. Innovative solutions for training such as video-based education in combination with box trainers should be promoted to mitigate the restrictions of face-to-face teaching	96%	
Research	30. Should clinical research restart?	91. Non-COVID clinical research should restart as soon as possible in line with safety recommendations and procedures	98%	
		92. Particular attention should be payed to research targeting preventive and mitigation strategies of aerosol contamination in the OR and safety of MIS	96%	

Initial disagreement was regarded priorities depending on local resources, the use of energy dissection devices and the risk of aerosolization, the indication for stenting for obstructed colorectal cancer, the indication of laparoscopic lavage for diverticular disease, the indication of neoadjuvant treatment for early rectal cancer in order to postpone surgery, the management of undiagnosed pancreatic lesions, the indication for intragastric balloons to postpone bariatric surgery and the indication for endoscopic therapies for achalasia and reflux disease to postpone surgery.

## Discussion

The study achieved its objective of formulating EAES evidence-based recommendations to provide guidance on the resumption of MIS across various general surgery specialities, taking into account the serious burden on our healthcare systems caused by the COVID-19 pandemic. These statements provided descriptive safety guidance measures that should be undertaken in the recovery plan for elective and emergency surgery across all subspecialties with specific measures for MIS.

In a recent survey, over 28 million patients are awaiting treatment worldwide, a number which continues to grow in the setting of new restrictions on delivery of care and a pandemic that is still evolving [24]. As this progression is expected to continue, and given the uncertainty about the ongoing pandemic, adaptive changes are required in procedure-based specialties to include safety, logistic, service relocation, economic and ethical considerations [25–30].

Through this project, consensus was achieved on all the proposed statements by the expert across the different domains, providing specific guidance on how to safely resume MIS and implement adaptive changes in procedure specific manner.

This project adhered to Delphi principles dictating anonymous voting. The selection of the steering group and the domains chairs was based on their peers’ recommendations and their leadership positions across different specialties and of their expertise on the research methodology of consensus development. The steering group did not contribute to the voting process.

The Delphi design allowed us to elicit the opinion of the EAES board members along with additional key stake holders to complement the multidisciplinary and heterogeneous nature of the international panel of experts. Although evidence synthesis was part of this project to generate evidence-based recommendations, there was no found evidence that can inform the statements, hence, relied on expert opinion.

A limited number of areas of continuing controversy were identified at the first voting round with lack of consensus among members. Initial disagreement was encountered on how to prioritize surgery, but ultimately total agreement was achieved by recommending that decisions should be based on local resources, the regional control of the COVID-19 pandemic and the patients’ medical condition. This was fundamental in deciding priorities also hereinafter for the rest of the consensus, but it is probably an area that requires fundamental research in the immediate future.

While there was also general agreement on the need to screen all patients undergoing surgery under general anaesthesia, by Reverse Transcription Polymerase Chain Reaction (RT-PCR) or even by computer tomography (CT) or ultrasound (US) lung scan in symptomatic patients, the option of regional anaesthesia should always be considered in suspected or positive patients for whom surgery cannot be postponed. Elective oncologic surgery should be only offered to SARS-CoV-2 negative patients or to previously positive patients after conversion to a negative RT-PCR COVID-19 test.

One of the main objectives of this project was to clarify the role of MIS, considering the conflicting information from different guidelines. This was based on the theoretical risk of possible contamination due to aerosolization and the gas leaks demonstrated during laparoscopy [31–33].

Until high-level evidence will be available to provide an answer about the direct link between SARS-CoV-2 contamination by pneumoperitoneum and its contagion to the operating team, the application of MIS across all surgical specialities has been supported by the experts in this project, provided local expertise is available and safety procedures are adhered to. Additionally, there was wide agreement that the general preference for MIS according to guidelines should not depend on the SARS-CoV-2 status or the indication of surgery in terms of elective and emergency settings.

These precautions are in line with the recent EAES/ SAGES recommendations to reduce gas leaks [34], the generation of smoke and by the use of surgical smoke evacuating systems [16]. These guidelines are also supported by the argument that containing potentially contaminated gas within a defined space, as it happens during laparoscopy, should provide a better control of risk, when compared to open surgery [11]. Regardless to the mode of surgery, limiting the use of energy devices in SARS-CoV-2 positive patients was also recommended and favouring ligatures/clips and/or stapling devices when possible. This is also in line with other recommendations (EAES/SAGES) and with evidence suggesting gas escape through trocars [30–32]. General recommendations for personnel safety in the OR including characteristics of the environment and PPE were also confirmed [16].

In the emergency setting, a number of recommendations were proposed by the experts, supporting conservative treatment for abscesses and collections in SARS-CoV-2 positive patients, rather than offering immediate surgery, if the general condition of patients allows this. On the contrary, for acute cholecystitis in SARS-CoV-2 positive patients, cholecystectomy was recommended when not responsive within 24 h rather than interventional treatment such as percutaneous transhepatic drainage, with the exception of ASA 3 and 4 patients.

In the field of abdominal wall surgery, laparoscopy was recommended for incarcerated ventral and inguinal hernia if not otherwise contraindicated and should not be postponed if clinically indicated. Emergency endoscopy (diagnostic and therapeutic) was supported in SARS-CoV-2 positive patients, as the first line to assess and possibly to treat bleeding, neoplastic obstruction, perforation and anastomotic leak. Similarly, laparoscopic surgery should be considered after failure of conservative/endoscopic management in symptomatic patients, as well as the in acute diverticulitis management according to the accepted algorithms.

Elective surgery for both malignant and benign disease should be postponed in SARS-CoV-2 positive patients until they return negative. This is also the case for hepatobiliary and pancreatic non-neoplastic diseases, as well as other oncologic patients, in whom interim procedures should be offered instead. For instance, drainage of the biliary tract by Percutaneous Transhepatic Biliary Drainage (PTBD) or Endoscopic Retrograde Cholangio Pancreatography (ERCP) should be considered as a bridging therapy.

In the field of bariatric surgery, the expert group in this project supported postposing elective surgery until the recovery plan, and flexible endoscopic procedures such as intragastric balloons was recommended as a bridge to surgery during the COVID-19 pandemic. Elective laparoscopic treatment for ventral and inguinal hernia in SARS-CoV-2 negative patients may need to be postponed depending on the local situation. Endocrine surgery should only be cancelled in SARS-CoV-2 positive patients until they convert to negative. Otherwise, patients should be prioritized depending on symptoms and oncological risk.

For upper and lower gastrointestinal cancer, neoadjuvant therapy could be considered for early cancers in order to postpone surgery after the COVID-19 pandemic, but only within registered studies, although difficult to arrange in short time. Functional disorders such as achalasia and reflux disease should be treated as usual and surgery can be considered if not responding to conservative treatment. The experts recommend that surgery should be delayed only in SARS-CoV-2 positive patients. Similarly, elective surgical treatment of benign colorectal pathologies should be prioritized based on patient and disease characteristics, local COVID-19 burden and institutional and staff resources. If not otherwise contraindicated, colorectal resections should be completed with anastomosis, while stoma formation should be applied as usual only for high-risk patients. Particular attention should be paid when Transanal Endoscopic Microsurgery (TEMS) or TransAnal Minimally Invasive Surgery (TAMIS) procedures are indicated, including TransAnal Total Mesorectal Excision (TaTME), due to the particularly high risk of operator contamination. In fact, alternative strategies for low rectal lesions, such as endoscopic mucosal resection and endoscopic submucosal dissection, should be considered in SARS-CoV-2 positive. On the other hand, due to its well proved benefits, a minimally invasive approach should be considered to treat colorectal cancer as well as benign conditions such as inflammatory bowel diseases and recurrent diverticulitis.

Finally, we focused on technology, education and research in the time of pandemic. The team of experts outlined how research activity on digital technology and robotics should be encouraged to focus on reducing the numbers of working personnel in wards, intensive care unit and the operating rooms. In fact, this pandemic highlighted the importance of technology advancement in remote teaching and mentorship. Innovative solutions for training such as video-based education in combination with box trainers should be promoted to mitigate the restrictions of face-to-face teaching. The experts outlined certain areas of further research targeting robotics, Artificial Intelligence, advanced imaging and energy devices that could have a positive impact in times of pandemic and restrictions due to social distancing. At the same time practical technological solutions including sustainable materials and steam sterizilation for PPE should be investigated in order to minimize production of waste.

Overall, this project highlighted interesting trends and controversies related to surgeons’ willingness to overcome this difficult time, but it holds a number of limitations. There is a lack of empirical data to support many of the underlying statements, hence weaknesses inherent to these guidelines include the reliance on expert opinion and discussion to formulate recommendations. Despite the limited evidence, this project highlighted a number of clinically relevant questions that provide an agenda to stimulate future research in this field. The selection of experts is another critical aspect within consensus statements development. The expert group involved in this research were all the EAES board members representing the research, technology and educational committees as well as the members of the executive committee of the society. The response rate among the participants was high across the entire process reflecting the hard work and commitment of the board members to undertake this project and complete the project in a timely manner given the urgency and the need for the guidelines.

## Conclusion

The recommendations formulated by the EAES board create a framework for resumption of surgery following COVID-19 pandemic with particular focus on the role of MIS across all specialities. The statements have the potential for wide application in clinical setting, education and research across different healthcare systems.

## Electronic supplementary material

Below is the link to the electronic supplementary material.

Supplementary file1 (DOCX 22 kb)
