# Supplementary material for: EAES Recommendations for Recovery Plan in Minimally Invasive Surgery Amid COVID-19 Pandemic
Source: Surg Endosc. 2020 Nov 10;35(1):1–17. doi: 10.1007/s00464-020-08131-0 (PMC7653984; doi:10.1007/s00464-020-08131-0)
Supplement: Supplementary file 1 — Supplementary file1 (DOCX 22 kb) [file 464_2020_8131_MOESM1_ESM.docx]

Appendix 1 - References used to generate statements and recommendations for all topics

1. Moletta L, Pierobon ES, Capovilla G, et al. International guidelines and recommendations for surgery during Covid-19 pandemic: A Systematic Review. *Int J Surg*. 2020;79:180-188. doi:10.1016/j.ijsu.2020.05.061
2. de Leeuw RA, Burger NB, Ceccaroni M, et al. COVID-19 and Laparoscopic Surgery: Scoping Review of Current Literature and Local Expertise. *JMIR Public Health Surveill*. 2020;6(2):e18928. Published 2020 Jun 23. doi:10.2196/18928
3. Thamboo A, Lea J, Sommer DD, et al. Clinical evidence based review and recommendations of aerosol generating medical procedures in otolaryngology - head and neck surgery during the COVID-19 pandemic. *J Otolaryngol Head Neck Surg*. 2020;49(1):28. Published 2020 May 6. doi:10.1186/s40463-020-00425-6
4. De Simone B, Chouillard E, Di Saverio S, et al. Emergency surgery during the COVID-19 pandemic: what you need to know for practice. *Ann R Coll Surg Engl*. 2020;102(5):323-332. doi:10.1308/rcsann.2020.0097
5. Spolverato G, Capelli G, Restivo A, et al. The management of surgical patients during the coronavirus disease 2019 (COVID-19) pandemic. *Surgery*. 2020;168(1):4-10. doi:10.1016/j.surg.2020.04.036
6. Welsh Surgical Research Initiative (WSRI) Collaborative. Recommended operating room practice during the COVID-19 pandemic: systematic review [published online ahead of print, 2020 May 12]. *BJS Open*. 2020;10.1002/bjs5.50304. doi:10.1002/bjs5.50304
7. Jessop ZM, Dobbs TD, Ali SR, et al. Personal Protective Equipment (PPE) for Surgeons during COVID-19 Pandemic: A Systematic Review of Availability, Usage, and Rationing [published online ahead of print, 2020 May 12]. *Br J Surg*. 2020;10.1002/bjs.11750. doi:10.1002/bjs.11750
8. Hirschmann MT, Hart A, Henckel J, Sadoghi P, Seil R, Mouton C. COVID-19 coronavirus: recommended personal protective equipment for the orthopaedic and trauma surgeon [published correction appears in Knee Surg Sports Traumatol Arthrosc. 2020 Jun 12;:]. *Knee Surg Sports Traumatol Arthrosc*. 2020;28(6):1690-1698. doi:10.1007/s00167-020-06022-4
9. Givi B, Moore MG, Bewley AF, et al. Advanced head and neck surgery training during the COVID-19 pandemic. *Head Neck*. 2020;42(7):1411-1417. doi:10.1002/hed.26252
10. Welsh Surgical Research Initiative (WSRI) Collaborative. Surgery during the COVID-19 pandemic: operating room suggestions from an international Delphi process [published online ahead of print, 2020 May 12]. *Br J Surg*. 2020;10.1002/bjs.11747. doi:10.1002/bjs.11747
11. Culp BM, Frisch NB. COVID-19 Impact on Young Arthroplasty Surgeons. *J Arthroplasty*. 2020;35(7S):S42-S44. doi:10.1016/j.arth.2020.04.058
12. Fontanella MM, De Maria L, Zanin L, et al. Neurosurgical Practice During the Severe Acute Respiratory Syndrome Coronavirus 2 (SARS-CoV-2) Pandemic: A Worldwide Survey. *World Neurosurg*. 2020;139:e818-e826. doi:10.1016/j.wneu.2020.04.204
13. Liebensteiner MC, Khosravi I, Hirschmann MT, Heuberer PR; Board of the AGA-Society of Arthroscopy and Joint-Surgery, Thaler M. Massive cutback in orthopaedic healthcare services due to the COVID-19 pandemic. *Knee Surg Sports Traumatol Arthrosc*. 2020;28(6):1705-1711. doi:10.1007/s00167-020-06032-2
14. Gruttadauria S; Italian Board of Experts in Liver Transplantation (I-BELT) Study Group, The Italian Society of Organ Transplantation (SITO). Preliminary Analysis of the Impact of the Coronavirus Disease 2019 Outbreak on Italian Liver Transplant Programs. *Liver Transpl*. 2020;26(7):941-944. doi:10.1002/lt.25790
15. Ota I, Asada Y. The impact of preoperative screening system on head and neck cancer surgery during the COVID-19 pandemic: Recommendations from the nationwide survey in Japan. *Auris Nasus Larynx*. 2020;47(4):687-691. doi:10.1016/j.anl.2020.05.006
16. Thaler M, Khosravi I, Hirschmann MT, et al. Disruption of joint arthroplasty services in Europe during the COVID-19 pandemic: an online survey within the European Hip Society (EHS) and the European Knee Associates (EKA). *Knee Surg Sports Traumatol Arthrosc*. 2020;28(6):1712-1719. doi:10.1007/s00167-020-06033-1
17. Grassi A, Pizza N, Tedesco D, Zaffagnini S. The COVID-19 outbreak in Italy: perspectives from an orthopaedic hospital. *Int Orthop*. 2020;44(8):1543-1547. doi:10.1007/s00264-020-04617-7
18. Jean WC, Ironside NT, Sack KD, Felbaum DR, Syed HR. The impact of COVID-19 on neurosurgeons and the strategy for triaging non-emergent operations: a global neurosurgery study. *Acta Neurochir (Wien)*. 2020;162(6):1229-1240. doi:10.1007/s00701-020-04342-5
19. Tan KA, Thadani VN, Chan D, Oh JY, Liu GK. Addressing Coronavirus Disease 2019 in Spine Surgery: A Rapid National Consensus Using the Delphi Method via Teleconference. *Asian Spine J*. 2020;14(3):373-381. doi:10.31616/asj.2020.0224
20. Ducournau F, Arianni M, Awwad S, et al. COVID-19: Initial experience of an international group of hand surgeons. *Hand Surg Rehabil*. 2020;39(3):159-166. doi:10.1016/j.hansur.2020.04.001
21. Amparore D, Claps F, Cacciamani GE, et al. Impact of the COVID-19 pandemic on urology residency training in Italy. *Minerva Urol Nefrol*. 2020;72(4):505-509. doi:10.23736/S0393-2249.20.03868-0
22. Boyarsky BJ, Po-Yu Chiang T, Werbel WA, et al. Early impact of COVID-19 on transplant center practices and policies in the United States. *Am J Transplant*. 2020;20(7):1809-1818. doi:10.1111/ajt.15915
23. Sahu D, Agrawal T, Rathod V, Bagaria V. Impact of COVID 19 lockdown on orthopaedic surgeons in India: A survey. *J Clin Orthop Trauma*. 2020;11(Suppl 3):S283-S290. doi:10.1016/j.jcot.2020.05.007
24. Gaudino M, Chikwe J, Hameed I, Robinson NB, Fremes SE, Ruel M. Response of Cardiac Surgery Units to COVID-19: An Internationally-Based Quantitative Survey. *Circulation*. 2020;142(3):300-302. doi:10.1161/CIRCULATIONAHA.120.047865
25. Allevi F, Dionisio A, Baciliero U, et al. Impact of COVID-19 epidemic on maxillofacial surgery in Italy. *Br J Oral Maxillofac Surg*. 2020;58(6):692-697. doi:10.1016/j.bjoms.2020.04.035
26. Vining CC, Eng OS, Hogg ME, et al. Virtual Surgical Fellowship Recruitment During COVID-19 and Its Implications for Resident/Fellow Recruitment in the Future [published online ahead of print, 2020 May 18]. *Ann Surg Oncol*. 2020;1-5. doi:10.1245/s10434-020-08623-2
27. Brown TS, Bedard NA, Rojas EO, et al. The Effect of the COVID-19 Pandemic on Electively Scheduled Hip and Knee Arthroplasty Patients in the United States. *J Arthroplasty*. 2020;35(7S):S49-S55. doi:10.1016/j.arth.2020.04.052
28. Porreca A, Colicchia M, D'Agostino D, et al. Urology in the Time of Coronavirus: Reduced Access to Urgent and Emergent Urological Care during the Coronavirus Disease 2019 Outbreak in Italy. *Urol Int*. 2020;104(7-8):631-636. doi:10.1159/000508512
29. Maffia F, Fontanari M, Vellone V, Cascone P, Mercuri LG. Impact of COVID-19 on maxillofacial surgery practice: a worldwide survey. *Int J Oral Maxillofac Surg*. 2020;49(6):827-835. doi:10.1016/j.ijom.2020.04.015
30. Torzilli G, Viganò L, Galvanin J, et al. A Snapshot of Elective Oncological Surgery in Italy During COVID-19 Emergency: Pearls, Pitfalls, and Perspectives. *Ann Surg*. 2020;272(2):e112-e117. doi:10.1097/SLA.0000000000004081
31. Nair AG, Gandhi RA, Natarajan S. Effect of COVID-19 related lockdown on ophthalmic practice and patient care in India: Results of a survey. *Indian J Ophthalmol*. 2020;68(5):725-730. doi:10.4103/ijo.IJO_797_20
32. Cai Y, Jiam NT, Wai KC, Shuman EA, Roland LT, Chang JL. Otolaryngology Resident Practices and Perceptions in the Initial Phase of the U.S. COVID-19 Pandemic [published online ahead of print, 2020 May 5]. *Laryngoscope*. 2020;10.1002/lary.28733. doi:10.1002/lary.28733
33. Latz CA, Boitano LT, Png CYM, et al. Early vascular surgery response to the COVID-19 pandemic: Results of a nationwide survey [published online ahead of print, 2020 May 23]. *J Vasc Surg*. 2020;S0741-5214(20)31287-8. doi:10.1016/j.jvs.2020.05.032
34. van Heijningen I, Frank K, Almeida F, et al. ﻿EASAPS/ESPRAS Considerations in getting back to work in Plastic Surgery with the COVID-19 Pandemic - A European point of view. EASAPS/ESPRAS-Überlegungen zur Rückkehr in den plastisch-chirurgischen Alltag während der COVID-19-Pandemie – eine europäische Sichtweise. *Handchir Mikrochir Plast Chir*. 2020;52(4):257-264. doi:10.1055/a-1175-4169
35. Luc JGY, Archer MA, Arora RC, et al. The Thoracic Surgery Social Media Network Experience During the COVID-19 Pandemic [published online ahead of print, 2020 May 18]. *Ann Thorac Surg*. 2020;10.1016/j.athoracsur.2020.05.006. doi:10.1016/j.athoracsur.2020.05.006
36. Oba A, Stoop TF, Löhr M, et al. Global Survey on Pancreatic Surgery During the COVID-19 Pandemic. *Ann Surg*. 2020;272(2):e87-e93. doi:10.1097/SLA.0000000000004006
37. Giunta RE, Frank K, Costa H, et al. The COVID-19 Pandemic and its Impact on Plastic Surgery in Europe - An ESPRAS Survey. Die COVID-19-Pandemie und ihre Auswirkungen auf die Plastische Chirurgie in Europa – Eine ESPRAS Übersicht. *Handchir Mikrochir Plast Chir*. 2020;52(3):221-232. doi:10.1055/a-1169-4443
38. Soriano Sánchez JA, Perilla Cepeda TA, Zenteno M, et al. Early Report on the Impact of COVID-19 Outbreak in Neurosurgical Practice Among Members of the Latin American Federation of Neurosurgical Societies. *World Neurosurg*. 2020;140:e195-e202. doi:10.1016/j.wneu.2020.04.226
39. Claps F, Amparore D, Esperto F, et al. Smart learning for urology residents during the COVID-19 pandemic and beyond: insights from a nationwide survey in Italy [published online ahead of print, 2020 May 20]. *Minerva Urol Nefrol*. 2020;10.23736/S0393-2249.20.03921-1. doi:10.23736/S0393-2249.20.03921-1
40. Athey AG, Cao L, Okazaki K, et al. Survey of AAHKS International Members on the Impact of COVID-19 on Hip and Knee Arthroplasty Practices. *J Arthroplasty*. 2020;35(7S):S89-S94. doi:10.1016/j.arth.2020.04.053
41. Haffer H, Schömig F, Rickert M, et al. Impact of the COVID-19 Pandemic on Orthopaedic and Trauma Surgery in University Hospitals in Germany: Results of a Nationwide Survey. *J Bone Joint Surg Am*. 2020;102(14):e78. doi:10.2106/JBJS.20.00756
42. Warth LC, Noiseux NO, Duncan ST, Daines SB, Mahoney CR. How Has COVID-19 Affected Our Orthopedic Implant Industry Partners? Implications for the Surgeon-Industry Relationship in 2020 and Beyond. *J Arthroplasty*. 2020;35(7S):S56-S59.e10. doi:10.1016/j.arth.2020.04.063
43. COVIDSurg Collaborative. Elective surgery cancellations due to the COVID-19 pandemic: global predictive modelling to inform surgical recovery plans [published online ahead of print, 2020 May 12]. *Br J Surg*. 2020;10.1002/bjs.11746. doi:10.1002/bjs.11746
44. Sud A, Jones ME, Broggio J, et al. Collateral damage: the impact on outcomes from cancer surgery of the COVID-19 pandemic. *Ann Oncol*. 2020;31(8):1065-1074. doi:10.1016/j.annonc.2020.05.009
45. Bedard NA, Elkins JM, Brown TS. Effect of COVID-19 on Hip and Knee Arthroplasty Surgical Volume in the United States. *J Arthroplasty*. 2020;35(7S):S45-S48. doi:10.1016/j.arth.2020.04.060
46. Jain A, Jain P, Aggarwal S. SARS-CoV-2 Impact on Elective Orthopaedic Surgery: Implications for Post-Pandemic Recovery [published online ahead of print, 2020 May 12]. *J Bone Joint Surg Am*. 2020;10.2106/JBJS.20.00602. doi:10.2106/JBJS.20.00602
47. Madanelo M, Ferreira C, Nunes-Carneiro D, et al. The impact of the coronavirus disease 2019 pandemic on the utilisation of emergency urological services. *BJU Int*. 2020;126(2):256-258. doi:10.1111/bju.15109
48. El-Hamamsy I, Brinster DR, DeRose JJ, et al. The COVID-19 Pandemic and Acute Aortic Dissections in New York: A Matter of Public Health. *J Am Coll Cardiol*. 2020;76(2):227-229. doi:10.1016/j.jacc.2020.05.022
49. Salenger R, Etchill EW, Ad N, et al. The Surge After the Surge: Cardiac Surgery Post-COVID-19 [published online ahead of print, 2020 May 4]. *Ann Thorac Surg*. 2020;S0003-4975(20)30693-7. doi:10.1016/j.athoracsur.2020.04.018
50. Ruthberg JS, Quereshy HA, Jella TK, et al. Geospatial analysis of COVID-19 and otolaryngologists above age 60. *Am J Otolaryngol*. 2020;41(4):102514. doi:10.1016/j.amjoto.2020.102514
51. Antony J, James WT, Neriamparambil AJ, Barot DD, Withers T. An Australian Response to the COVID-19 Pandemic and Its Implications on the Practice of Neurosurgery. *World Neurosurg*. 2020;139:e864-e871. doi:10.1016/j.wneu.2020.05.136
52. Pinar U, Anract J, Duquesne I, et al. Impact de la pandémie de COVID-19 sur l’activité chirurgicale au sein des services d’urologie de l’Assistance Publique – Hôpitaux de Paris [Impact of the COVID-19 pandemic on surgical activity within academic urological departments in Paris]. *Prog Urol*. 2020;30(8-9):439-447. doi:10.1016/j.purol.2020.05.001
53. Kiong KL, Guo T, Yao CMKL, et al. Changing practice patterns in head and neck oncologic surgery in the early COVID-19 era. *Head Neck*. 2020;42(6):1179-1186. doi:10.1002/hed.26202
54. Nuñez JH, Sallent A, Lakhani K, et al. Impact of the COVID-19 Pandemic on an Emergency Traumatology Service: Experience at a Tertiary Trauma Centre in Spain. *Injury*. 2020;51(7):1414-1418. doi:10.1016/j.injury.2020.05.016
55. Chow VLY, Chan JYW, Ho VWY, et al. Conservation of personal protective equipment for head and neck cancer surgery during COVID-19 pandemic. *Head Neck*. 2020;42(6):1187-1193. doi:10.1002/hed.26215
56. Morrison DR, Gentile C, McCammon S, Buczek E. Head and neck oncologic surgery in the COVID-19 pandemic: Our experience in a deep south tertiary care center. *Head Neck*. 2020;42(7):1471-1476. doi:10.1002/hed.26262
57. Bram JT, Johnson MA, Magee LC, et al. Where Have All the Fractures Gone? The Epidemiology of Pediatric Fractures During the COVID-19 Pandemic. *J Pediatr Orthop*. 2020;40(8):373-379. doi:10.1097/BPO.0000000000001600
58. Mehta AI, Chiu RG. COVID-19 Nonessential Surgery Restrictions and Spine Surgery: A German Experience. *Spine (Phila Pa 1976)*. 2020;45(14):942-943. doi:10.1097/BRS.0000000000003571
59. Cai M, Wang G, Zhang L, et al. Performing abdominal surgery during the COVID-19 epidemic in Wuhan, China: a single-centred, retrospective, observational study. *Br J Surg*. 2020;107(7):e183-e185. doi:10.1002/bjs.11643
60. von Dercks N, Körner C, Heyde CE, Theopold J. Wie stark trifft die Corona-Pandemie die Kliniken für Orthopädie und Unfallchirurgie? : Eine Analyse der ersten 5 Wochen [How badly is the coronavirus pandemic affecting orthopaedic and trauma surgery clinics? : An analysis of the first 5 weeks]. *Orthopade*. 2020;49(6):494-501. doi:10.1007/s00132-020-03926-4
61. Noureldine MHA, Pressman E, Krafft PR, et al. Impact of the COVID-19 Pandemic on Neurosurgical Practice at an Academic Tertiary Referral Center: A Comparative Study. *World Neurosurg*. 2020;139:e872-e876. doi:10.1016/j.wneu.2020.05.150
